# Anicteric hepatoxicity: a potential health risk of occupational exposures in Nigerian petroleum oil refining and distribution industry

**DOI:** 10.1186/1745-6673-9-3

**Published:** 2014-01-22

**Authors:** Tobias I Ndubuisi Ezejiofor, Anthonet N Ezejiofor, Orish E Orisakwe, Hariet C Nwigwe, Ferdinand OU Osuala, Moses OE Iwuala

**Affiliations:** 1 Department of Biotechnology, School of Science, Federal University of Technology, Owerri, Nigeria; 2 Department of Biological Science, School of Science, Federal University of Technology, Owerri, Nigeria; 3 Department of Clinical Pharmacy, Toxicology Unit, University of Port Harcourt, Port Harcourt, Nigeria

**Keywords:** Anicteric hepatoxicity, Occupational exposures, Petroleum refining and distribution industry, Nigeria

## Abstract

**Background:**

Literature abounds linking one’s job to certain unpalatable health outcomes. Since exposures to hazardous conditions in industrial environments often results in sundry health effects among workers, we embarked on this study to investigate the hepatic health effects of occupational activities in the petroleum refining and distribution industry.

**Method:**

Biochemical markers of liver functions were assayed in plasma, using Reflotron dry chemistry spectrophotometric system. The study was conducted on randomly selected workers of Port Harcourt Refining Company (PHRC) and Pipelines and Petroleum Product Marketing Company (PPMC) both in Alesa-Eleme near Port Harcourt, Nigeria, as well as non-oil work civil servants serving as control subjects.

**Result and conclusion:**

Results showed that, bilirubin ranged 0.3-1.6 mg/dl with a mean of 0.66±0.20mg/dl among the oil workers as against 0.5-1.00mg/dl with a mean of 0.58±0.13mg/dl in non-oil workers, Alkaline phosphatase ranged 50.00-296.00u/l (mean: 126.21±39.49u/l) in oil workers as against 40.20-111u/l (mean: 66.83±18.54u/l) for non-oil workers, Aspartic transaminases (AST) ranged 5.80-140.20u/l (mean: 21.81±11.49u/l) in oil workers against 18.00-44.00u/l (mean: 26.89±6.99u/l) for non-oil workers, while Alanine transaminases (ALT) ranged 4.90-86.00u/l (mean: 22.14±11.28u/l) in oil workers as against 10.00-86.60u/l (mean: 22.30±10.22u/l) for the non-oil workers. A close study of the results revealed that although the mean values for all the studied parameters were still within the parametric reference ranges, however, relative to the referents, there were significant increases (P<0.05) in plasma bilirubin (though anicteric) and alkaline phosphatase that was not matched with a corresponding increase in the plasma transaminases, suggesting a possibility that toxic anicteric hepatoxicity is part of the potential health effects of sundry exposures in the Nigeria petroleum oil refining and distribution industry. Gender differentiation data showed that though the mean values for the parameters were higher in males than females, the increases were not significant in most cases (P>0.05), whereas data for age and exposure period classifications revealed that irrespective of the age of the worker, the effects are likely to start after the first five years, manifesting fully after the first decade of occupational exposures. Thus, an update of industrial/occupational health measures is necessary for a safer and healthier work environment.

## Background

### Petroleum- components, ecological distribution and toxicological impacts

Petroleum consists of crude oils and a variety of refined oil products, and is also a significant source of polycyclic aromatic hydrocarbons (PAH) [1]. Pollution by petroleum hydrocarbons being widespread and frequent, can arise accidentally wherever oil is produced, transported, stored, processed, or used at sea or land. On land, petroleum products can account for a large proportion of the chemicals at contaminated sites [2]. Wilson and LeBlanc). Contamination of groundwater and soil by specific hydrocarbon compounds (e.g. benzene leaking from underground fuel storage tanks) has led many countries to enact regulatory requirements for action and cleanup of these compounds [3]. An estimated 2 to 3 million tons of petroleum hydrocarbons reach the world’s seas and oceans each year [4]. Municipal and industrial wastes, urban and river runoff account for a significant proportion of petroleum products in fresh water systems and in the marine environments. Oil refineries discharge waste water containing some petroleum hydrocarbons. The receiving waters are therefore subject to low level chronic pollution [2]. Due to its complicated composition, petroleum has the potential to elicit multiple types of toxic effects. It can cause acute lethal toxicity, sub-lethal chronic toxicity, or both depending on the exposure, dosage, and organism exposed. Some PAH components of petroleum have the potential to bioaccumulate within susceptible aquatic organisms and be passed by trophic transfer to other levels of the food chain [5,6]. Petroleum can also affect living organisms by other means such as through physical oiling and through alterations in their habitat. When an oil spill occurs, toxicities are not limited to acute effects restricted to the immediate vicinity of the discharge. Due to dispersion, dilution and deposition of oil into the water column and onto shores and into sediments via weathering and other mechanisms, all organisms within the area may be exposed to petroleum hydrocarbon components for an extended period of time. Crude oil and many of its individual components have been reported to cause a variety of some lethal effects in a wide array of living organisms. Some of the most frequently observed long-term petroleum pollution on individual organisms include impaired reproduction [7], reduced growth [5], tumours and lesions [8]), blood disorders [9], and morphological abnormalities [10]. Female rats exposed to petroleum hydrocarbons showed evidence of nephrotoxic effects [11].

### Petroleum refining and distribution industry: exposures and health implication

Petroleum refining and distribution are among the occupational activities perceived to be hazardous. Petroleum refining has evolved continuously in response to changing consumer demands for better and different products. Once extracted, crude oil is transported to an oil refinery where complex hydrocarbon compounds are separated and converted through various refining operations (fractional distillation, cracking, solvent extractions, then other treatments including formulating and blending) to become useable fuel sources. Finally, impurities are removed through chemical treatment of each product. The process of refining oil manufactures nearly 2,500 useful products [12]. The major end product of oil is gasoline, followed by diesel fuel, jet fuel, fuel oil, kerosene, lubricating oil and asphalt used for road paving. Apart from the products, oil refineries also contribute various forms of pollution including thermal and noise pollution. Thermal pollution involves the discharge of effluents that are significantly warmer than surrounding water, while noise level in refineries can exceed 90 decibels, posing a significant threat to the health and safety of oil refinery employees and even the surrounding community because leakage of noise pollution can have significant psychological effects on local residents, decreases aesthetics of the area and can interfere with wildlife [13].

Oil refinery workers are continuously exposed to numerous hazardous materials and working conditions that place them at continuous risk of injury and death. Such chronic hazards include exposure to noise, heat, polluted air, wide varieties of numerous hazardous chemical substances used either as process chemicals and/or present in resultant effluents/wastes, as well as in the usually invisible emissions, which include petroleum itself and other aromatic hydrocarbons (benzene, toluene, phenol, etc.), hydrogen sulphide and other natural gases (methane, propane, butane, etc.), carbon monoxide, asphalt, toxic heavy metals (arsenic, chromium, cadmium, nickel, zinc, etc.), coke dust, lead alkyls, silica, asbestos etc. [14,15]. Asphalt for example, can cause severe burns and eye irritation, and its fumes may contain unacceptable levels of benzene and hydrogen sulphide, which may lead to dermatitis, bronchitis and chemically induced pneumonia [16]. Continuous exposure to carbon monoxide can lead to headaches and mental disturbances, and at high concentrations may bring about death from asphyxiation. Long-term exposure to coke dust, silica and hydrogen sulphide can lead to chronic lung disease, while Lead alkyls used as gasoline additives can lead to psychosis and peripheral neuropathies. Asbestos, often used in oil refineries for the thermal insulation of boilers and pipes, has long been associated with pulmonary fibrosis, lung cancer and malignant mesothelioma among maintenance, repair and removal workers [16]. Even though the use of asbestos is banned in some countries, it is still widely used in many parts of the world. The use of asbestos in developing countries is a major problem as these nations often lack legislation regarding the use of asbestos, and the long-term health and safety of workers is often a low priority [12].

Oil refineries are inherently complex in their equipment and structural design that combined with the multitude of chemicals used, workers are at a continuous risk of accidents involving fires, explosions, chemical spills and burns as well as numerous health effects, many of which may remain latent even onto or post-retirement from active service. Accidental oil spills and leaks harm both workers and surrounding ecosystems because of exposure to petroleum and petroleum derivatives. Explosions and fire potentials stem from natural gases such as methane, propane, and butane produced at the refineries. Microscopic flaws in metal pipes fittings or fixtures in “hydro crackers” that operate at pressures ranging from 500 to 3200 pounds per square inch and temperatures ranging from 500 to 1800°f [14] can also lead to dangerous explosions. Accidental oil spills and leaks harm both workers and surrounding ecosystems because of exposure to petroleum and petroleum derivatives. Although oil spills most commonly occur during transport, leaking oil storage tanks and barrels contribute significantly to chemical and oil spills at refineries. Oil refineries and transport efforts account for approximately 46% of the estimated 3.2 million tons of oil entering the ocean each year [16]. The oil and oil waste products eventually find their way into the atmosphere, water column, bottom sediments and beaches. The resulting build-up of petroleum waste matter in an area has impact on surrounding biota and ecosystems [17].

### Operational area facilities, activities and products: potential sources of hazards

Alesa-Eleme near Port Harcourt, Nigeria, West Africa, is the location of the main operational facilities of both PHRC and PPMC, and lies within latitude 4.7706° and longitude 7.1056°. Petroleum refining and distribution industry in Nigeria, as elsewhere, constitute a giant industry with many complicated systems. It consists of two refineries in one: the old Port Harcourt refinery (Area 5), and the New Port Harcourt Refinery (Areas 1- 4). Both refineries have combined installed capacity of 210,000bpsd [18]. The PHRC has five process areas, areas1-5 each of which houses several operational units such as the following: crude distillation unit (CDU), saturated gas concentration unit (SGCU), vacuum distillation unit (VDU)- area1; Naphtha hydrotreating unit (NHU), catalytic reforming unit (CRU) with continuous catalyst regeneration (CCR) section, kerosene hydrotreating unit (KHU)-area2; fluid catalytic cracking unit (FCCU), gas concentration unit (GCU), gas treating unit (GTU), merox unit- area3; dimersol units, HF alkylation unit, butamer unit- area4; crude distillation unit (CDU) crude reforming unit (CRU) and liquefied petroleum gas (LPG) plant- area5. Beside these, the refinery also has a power plant and utility (PPU) section equipped with boilers and other facilities that generates its power and other utilities, as well as a waste water treatment (WWT) section, which treats refinery waste water before being discharged to the neighbouring creek. These units or sections are named according to the nature of activities or work performed in them, and accordingly, they also constitute potential sources of exposures to the workers performing those activities, and particularly so when the facilities or parts thereof become defective and consequent outlets to misty and gaseous fumes of various process materials, products or wastes/effluents. The process of production and the actual products are both sophisticated. The major end product of oil is gasoline, followed by diesel fuel, jet fuel, fuel oil, kerosene, lubricating oil and asphalt used for road paving. PHRC processes crude oil (Bonny Light) into liquefied petroleum gas (LPG), Premium Motor Spirit (PMS), Dual purpose Kerosene (DPK) (Aviation and Domestic), Automotive Gas Oil (AGO– Diesel), Low Pour Fuel Oil (LPFO), and High Pour Fuel Oil (HPFO) as well as many other intermediate products that are industrially and domestically very useful. Through complex network of pipelines and storage tanks, these products of the refineries are passed over to the Pipelines and Petroleum Marketing Company (PPMC) for subsequent distribution. Behind all these facilities and their operations are workers, who are therefore, considered liable to certain health effects and/or impacts on account of several hazards from sundry job exposures in the various work units. Unraveling the actual nature and extent of these health effects/impacts is the main thrust of this study.

The knowledge that most human diseases and sufferings are sometimes related to the hazards of their workplace meant that appropriate remedies to the situation would come only when these hazards are properly assessed, their very nature, extent and impacts firmly established. Consequently, the need for toxicological data regarding industrial exposures and their health effects has been variously expressed by industrial stakeholders and researchers. However, for most of the industrial establishments in Nigeria particularly the numerous small and medium scale industries that form the bedrock of her industrial activities, and even some of the large scale industrial concerns (inclusive of the petroleum and petrochemical industry that is the mainstream of her economy), such occupational studies for proper assessment/establishment of the hazards are yet to be carried out. Thus, there is need for concerted effort towards providing some of the industrial exposures-health effects data called for by Loewenson [19], and particularly Scala [20] who had emphasized a three-fold need for toxicological data on the part of petroleum or petrochemical industry. In the Nigerian setting, available data regarding petroleum industry are based on studies using animal models, but non on humans, who for occupational reasons are subjected to long term, low-level continuous exposures to petroleum fractions, intermediates and finished products, as well as other hazardous conditions in their work environments, as is the case with petroleum refining and distribution industry workers. To the best of our knowledge, it is most uncertain that these workers have been directly investigated for any health effects or toxicological impacts of their occupational activities in the industry. Consequently, there is dearth of data regarding the nature and extent of health effects of sundry job exposures in this industry in Nigeria. Studies done elsewhere with regard to workers in the oil and gas industry had documented some organs/systems health effects, producing various morbidities and mortalities [21-32]. Although previous Nigerian studies have reported the effects of petroleum exposures in animal models that could possibly be extrapolated for humans [33-39], there is need for a direct human assessment of the situation using human biological samples. Results to be obtained from such direct human studies is expected to give a more assured situation with regards to human toxicology of petroleum products than an extrapolated result, which might be affected by species differences.

Meanwhile, risk assessment of petroleum refining and distribution industrial work environment revealed that the workers are furnished with sundry hazardous exposures [40], just as a study of some anthropometrical and biochemical markers also showed cardiovascular diseases and toxic nephropathy as part of diverse health hazards of this work cohorts [41,42]. Presently, the impacts of these exposures, particularly as it concerns the hepatic systemic effects remain uncertain in Nigeria, and thus forms the major focus of this study aimed at exploring the implications of workplace conditions and exposures to a wide range of potentially hepatotoxic substances present in emissions in the environment of petroleum refining and distribution industry. These include petroleum itself, benzene, dioxins, furans, other aromatic hydrocarbons (toluene, phenol, etc.), hydrogen sulphide and other natural gases (methane, propane, butane, etc.), carbon monoxide, asphalt, toxic heavy metals (arsenic, hexavalent chromium, cadmium, nickel, zinc, etc.) [14,15], all of which have deleterious effects on human body, and have also been recognized as carcinogens by the International Agency for Research on Cancer (IARC) [8]. In relation to their metabolic functions, the liver in humans, animal models or naturally exposed fauna, are target organs for several substances and metals (at least Cd or As), and accumulations of these beyond its metabolic detoxification capacity portend serious dangers to the hepatic health, and by extension, the overall health of the individual. Meanwhile, data from the plant clinic jointly used by the two establishments being studied revealed liver diseases as making reckonable contribution to the morbidities and mortalities recorded in this industry [43,44], suggesting that these diseases might be prevalent among the workers, and thus warranting further studies. Here lies the justification for the present evaluation of hepatic health risk among workers of this sector, using relevant biomarkers.

### Objective of the study

This study hopes to contribute towards providing some of the necessary data called for by Loewenson [19] and in particular, Scala [20] who had emphasized a three-fold need for toxicological data on the part of petroleum or petrochemical industry. In the Nigerian setting, available data are based on studies using non-human animal species as can be gleaned from the studies cited above, but definitely non-existent for the humans who for occupational reasons are subjected to long term, low-level continuous exposures to petroleum fractions, intermediates and finished products, as well as other hazardous conditions in their work environments, as may be the case with petroleum refining and distribution industry workers. Although most toxicological studies conducted with respect to petroleum industry in the Nigerian environment had reported some biological effects of petroleum products on the animal models used, it is most uncertain that workers of this industrial sector have been directly investigated for any health effects or toxicological impacts of their occupational activities in the petroleum refining and distribution industry. Consequently, there is dearth of data regarding the nature and extent of health effects of occupational exposures in this industry in the Nigerian setting. This forms the scope of what this study seeks to address. Thus, the objective of the study is to evaluate these workers for possible health effects of occupational exposures in this industry, though with particular focus on the hepatic system, and to determine the role of gender, age and exposure period in defining any observed effect. Since our present attention is on the hepatic system, liver function markers would be assayed in the biological samples to be provided by the study participants. The outcome of this research would among other things:

1. clear the uncertainties about the dangers of occupational exposures in the petroleum refining and distribution industrial sector,

2. provide a firmer ground to sue (or not to sue) for health compensation for the retired/disengaged or estranged workers who may have already fallen victims of various organic ailments, particularly hepatic disease conditions, following several years of service in this sector, and

3. provide data-based evidence for update of industrial/occupational health measures aimed at improving health of workers and eventually their productivity

## Materials and methods

### Subjects

The participants of this study consisted of three hundred and thirty three (333) human subjects aged between 28 and 60 years old. Of this number, three hundred and three (303) study participants (273 males and 30 females) were randomly drawn from the staff of the two industrial establishments studied: Port Harcourt Refining Company Ltd (PHRC) (Petroleum refiners) and Pipelines and Petroleum Products Marketing Company (PPMC) (Petroleum distributors); the remaining 30 were non-oil sector civil servants considered healthy (20 males and 10 females) also randomly recruited to serve as referents or comparison group, and these were mainly classroom teachers in institutions of higher learning. The nature and purpose of the study was explained to the participants, following which they willingly consented to participation in the study.

### Ethical clearance

Ethical Clearance was obtained from the Institutional Review Committee of the Department of Petroleum Resources (DPR), Federal Ministry of Petroleum Resources (the supervisory ministry). The nature and purpose of the study was explained to the management and staff of the establishments studied, following which approval for study and consent for voluntary participation respectively were obtained.

### Inclusion and exclusion criteria

For the study participants from the industry, only those on the job for a period not less than 3 years (service period of 3 years or more) were included, while those with less service period were excluded. For the referents, exclusion criteria were involvement(s) in petroleum refining and distribution activities or any other activity that warrants prolonged and close contact/exposure to petroleum and gas products. Based on these criteria, 27 oil workers and 3 Non oil workers were excluded.

### Sample

Using syringes and needles, blood (5 ml) was collected from each of the participants and dripped into a Lithium heparin anticoagulant specimen container. The sample was gently but thoroughly mixed to ensure the blood did not clot. The heparinized tube sample was subsequently centrifuged and the resultant plasma separated from the cells and then stored in the refrigerator for subsequent hepatic biochemical profile assays.

### Methodology/data analysis

The biochemical markers of liver function determined include plasma bilirubin, Alkaline phosphatase, Aspartic and Alanine transaminases. The resultant data were analyzed using statistical programme for social sciences (SPSS) Version11. Descriptive statistics, student’s T-test, ANOVA, multiple comparisons analyses using least significant difference (LSD) (Post Hoc tests), regression and correlation analyses were some of the statistical tools adopted for analyses.

### Determination of plasma total Bilirubin

Plasma total bilirubin assay was performed based on the reaction principle of Freitag et al. [45]. After application of the sample (Plasma) to the Bilirubin test strip (made by Roache Diagnostic GmbH (Germany), the sample flows into the reaction zone. Before the beginning of reaction however, the protein-bound indirect bilirubin is released by means of dyphylline [7-(2, 3-dihydroxypropyl) – theophyline], so that both the direct and indirect bilirubin react with the diazonium salt, 2-methoxy-4-nitrophenyldiazonium-tetrafluoroborate Bilirubin + 2-methoxy-4-nitrophenyldiazonium tetrafluoroborate Azobilirubin.

At a temperature of 37°C, the dye formed is displayed after about 135 seconds in mg/dl.

### Determination of plasma aspartic transaminase (AST), formerly glutamate oxaloacetate transaminase (GOT) activity

The assay of this enzyme activity was done based on the reaction principle of Deneke et al. [46] using the Reflotron dry chemistry spectrophotometric system. After application to the AST Test Strip (made by Roache Diagnostic GmbH (Germany), the sample flows into the reaction zone. In the presence of GOT, ∝ − ketoglutarate and Alanine sulfinate are converted to pyruvate and glutamate. In a second reaction step catalyzed by pyruvate Oxidase (PyOD), the resulting pyruvate is cleaved into acetyl phosphate, carbon dioxide and hydrogen peroxide. In the presence of peroxidase (POD) the hydrogen peroxide converts an indicator into its oxidized blue form:

$$\begin{array}{ll} \;\propto -\text{Ketoglutarata} & +\text{alanine}\;\text{sulfinate}{\left(\text{Aspartate}\right)}^{{}^{\text{GOT}}}\;\text{glutamata}\\ & +SO_{3}^{2-} \end{array}$$

$$\begin{array}{ll} \;\text{Pyruvate} & +\text{PO}4^{3-}+O^{2-}+H_{2}O^{\text{PyOD}}\;\text{acetylphosphate}\\ & +H_{2}O_{2}+CO_{2} \end{array}$$

$$H_{2}O_{2}+\text{Indicator}{\left(\text{red}\right)}^{\text{POD}}\;\text{indicator}\left(\text{ox}\right)+H_{2}O$$

Endogenous Pyruvate is eliminated in a preliminary reaction. At a temperature of 37°C, the formation of the dye is measured kinetically at 567 nm as a measure of the enzyme activity of GOT and the result displayed after 124 seconds in U/L.

### Determination of plasma Alanine transaminase (ALT), formerly glutamate pyruvate transaminase (GPT) activity

The assay of this enzyme activity was done based on the principle of Deneke and Rittersdorf [47], using the Reflotron dry chemistry spectrophotometric system. After application to the ALT Test Strip (made by Roache Diagnostic GmbH (Germany), the sample flows into the reaction zone. In the presence of ALT (GPT), ∝ − ketoglutarate and Alanine are converted to glutamate and pyruvate. In a second reaction step catalyzed by pyruvate oxidase, the resulting pyruvate is cleaved into acetyl phosphate, carbon dioxide and hydrogen peroxide. In the presence of POD, the hydrogen peroxide converts an indicator into its oxidized blue form:

$$\propto -\text{Ketoglutarate}+\text{alanin}e^{\text{GPT}}\;\text{glutamate}+\text{pyruvate}$$

$$\begin{array}{ll} \;\text{Pyruvate} & +P{O_{4}}^{2-}+O^{2-}+H_{2}O^{\text{PYOD}}\;\text{acetylphosphate}\\ & +H_{2}O_{2}+CO_{2} \end{array}$$

$$H_{2}O_{2}+\text{Indicator}{\left(\text{red}\right)}^{\text{POD}}\;\text{indicator}\left(\text{ox}\right)+H_{2}O$$

Endogenous Pyruvate is eliminated in a preliminary reaction. At a temperature of 37°C, the formation of the dye is measured kinetically at 567 nm as a measure of the enzyme activity of GPT and the result displayed after 140 seconds in U/L.

### Determination of plasma alkaline phosphatase (ALP) activity

The assay of this enzyme activity was done based on the reaction principle of Heins et al. [48] using the Reflotron dry chemistry spectrophotometric system. After application to the ALP Test Strip (made by Roache Diagnostic GmbH (Germany), the sample flows into the reaction zone. ALP hydrolyzes O-cresolphthalein phosphate to O-cresolphthalein and transfers the phosphate group to the acceptor molecule methylglucamine. The coloured hydrolysis product, O-cresolphthalein that is produced per unit of time under alkaline conditions is directly proportional to alkaline phosphatase activity:

$$\begin{array}{ll} \;O- & \text{cresolphthalein}\;\text{phosphate}\\ & +\;\text{methylglucamin}e^{\text{ALP}}\;O-\text{cresolphthalein}\\ & +\;\text{methylglucamine}\;\text{phosphate}. \end{array}$$

At a temperature of 37°C, the formation of the coloured reaction product is measured kinetically at 567 nm as a measure of the enzyme activity of ALP and the result displayed after 135 seconds in U/L.

## Results

The results of the hepatic function markers studied in the oil workers and referents are summarized in the following tables:

Table 1 reported on the ranges and mean values of hepatic function markers studied in the oil workers (PPMC and PHRC put together) as compared with those of the non-oil workers (referents). A close look at these values showed that despite a significantly lower mean age of oil workers relative to that of their non-oil work counterpart (43.3±7.03 yrs vs. 47.37±6.74 yrs, P < 0.01) and a significantly lower mean value for service (exposure) period in the oil workers than non-oil workers (16.18 ±6.42 yrs vs. 20.03±8.77 yrs, P < 0.01), the mean values of the studied health markers were significantly higher in oil workers for plasma Total bilirubin (0.66±0.20 mg/dl vs. 0.58±0.13 mg/dl, P < 0.01) and ALP (126.21±39.49u/l vs. 66.83±18.54u/l) but lower in oil workers than the referents for AST (21.81±11.49u/l vs. 26.89±6.99u/l, P < 0.05) and ALT (22.14±11.28u/l vs. 22.30±10.22u/l, P > 0.05), with the difference in AST being significant while that of ALT was not. Also, the oil workers and their control counterparts were separated according to gender and age groupings, and the mean values of studied parameters for these groupings were as presented in Tables 2 (Males), Table 3 (Females), and Table 4 (Age groups). For these groupings, the presentation pattern was as earlier observed, however, though the mean values for the parameters were higher in males than females, the increases were not significant in most cases (P > 0.05).

**Table 1:** Value ranges and means of liver function parameters studied in the oil workers (PPMC and PHRC put together) compared with the NON-OIL WORKERS

**Parameter**	**Non-oilworkers (N = 30)**	**Mean ± SD**	**Oil workers (N = 303)**	**Mean ± SD**	**P-value (2-tailed)**	**Normal range & unit of parameter**
	**Range**		**Range**			
**Age**	**35.00-59.00**	47.37 ± 6.74*	**28.00-60.00**	**43.30 ± 7.03**	**0.003**	
Service period**	**3.00-34.00**	20.03 ± 8.77*	**3.00-34.00**	**16.18 ± 6.42**	**0.003**	**Up to 35 yrs**
**Total bilirubin**	**0.50-1.00**	**0.58 ± 0.13**	**0.30-1.60**	0.66 ± 0.20*	**0.000**	**0- 1.00 mg/dl**
**ALK. PHOS**	**40.00-111.00**	**66.83 ±18.54**	**50.00-296.00**	126.21 ± 39.49*	**0.000**	**50-250 U/L**
**AST**	**18.00-44.00**	26.89 ± 6.99*	**5.80-140.20**	**21.81 ± 11.49**	**0.018**	**0-45 U/L (M), 0-35 U/L (F)**
**ALT**	**10.00-55.00**	**22.30 ± 10.22**	**4.90-86.60**	**22.14 ± 11.28**	**0.941**	**0-45 U/L (M), 0-35 U/L (F)**

T-test was the statistical tool applied. * indicates statistically significant difference [**Service Period = number of years the worker had been engaged in the work, amounting to his/her cumulative years of exposure to the hazards of his/her workplace (i.e. exposure period). Presently, 35 yrs is the maximum service period that qualifies an average civil servant for retirement in Nigeria, and is therefore considered as the upper limit of normal for service period in this report].

**Table 2:** Mean values of liver function parameters studied in male oil workers & non-oil male workers

**Parameter**	**Non-oilworkers (N = 20)**	**Oil workers (N = 273)**	**P-value (2-tailed)**	**Normal range & unit of parameter**
	**Mean ± SD**	**Mean ± SD**		
**Age**	49.75 ± 5.15*	**43.63 ± 6.98**	**0.000**	
**Service period**	23.20 ± 7.81*	**16.32 ± 6.47**	**0.000**	**Up to 35 yrs**
**Total bilirubin**	**0.60 ± 0.14**	**0.67 ± 0.20**	**0.124**	**0- 1.00 mg/dl**
**ALK. PHOS**	**65.90 ± 19.56**	126.69 ± 39.57*	**0.000**	**50-250 U/L**
**AST**	28.02 ± 6.71*	**22.25 ± 11.88**	**0.033**	**0-45 U/L (M), 0-35 U/L (F)**
**ALT**	**22.80 ±9.00**	**22.55 ± 11.51**	**0.925**	**0-45 U/L (M), 0-35 U/L (F)**

T-test was the statistical tool applied. *indicates statistically significant difference.

**Table 3:** Mean values of liver function parameters studied in female oil workers & non-oil female workers

**Parameter**	**Non-oilworkers (N = 10)**	**Oil workers (N = 30)**	**P-value (2-tailed)**	**Normal range & unit of parameter**
	**Mean ± SD**	**Mean ± SD**		
**Age**	**42.6 ± 7.25**	**40.33 ± 6.9**	**0.379**	
**Service period**	**13.7 ± 7.21**	**14.97 ± 5.84**	**0.579**	**Up to 35 yrs**
**Total bilirubIin**	**0.54 ± 0.07**	0.61 ± 0.17*	**0.228**	**0- 1.00 mg/dl**
**ALK. PHOS**	**68.7 ± 17.13**	**121.87 ± 39.16**	**0.000**	**50-250 U/L**
**AST**	24.63 ± 7.34*	**17.73 ± 5.67**	**0.004**	**0-45 U/L (M), 0-35 U/L (F)**
**ALT**	**21.3 ± 12.79**	**18.41 ± 8.15**	**0.408**	**0-45 U/L (M), 0-35 U/L (F)**

T-test was the statistical tool applied. *indicates statistically significant difference.

**Table 4:** Mean values of liver function parameters studied for the various age groups of the oil workers & control subjects

**Age group**	**Parameter**	**Non-oilworkers Mean ± SD**	**Oil workers Mean ± SD**	**P-value**
**30-39 (Non-oil workers, n = 5), (Oil workers, n = 97)**	**Age**	**36.60 ± 1.52**	**35.55 ± 2.69**	**0.651**
	**Service period**	**10.00 ± 4.18**	11.46 ± 4.34*	**0.000**
	**Total bilirubin**	**0.52 ± 0.04**	0.66 ± 0.18*	**0.035**
	**ALK. PHOS**	**62.60 ± 19.83**	133.41 ± 40.17*	**0.000**
	**AST**	**23.00 ± 2.09**	**22.59 ± 9.17**	**0.066**
	**ALT**	**16.78 ± 6.30**	**24.02 ± 12.99**	**0.462**
**40-49 (Non-oil workers, n = 13), (Oil workers, n = 142)**	**Age**	**45.46 ± 2.60**	**44.27 ± 2.71**	
	**Service period**	**19.62 ± 7.30**	16.82 ± 5.34*	**0.005**
	**Total bikirubin**	**0.56 ± 0.09**	0.66 ± 0.20*	**0.041**
	**ALK. PHOS**	**66.23 ± 12.40**	121.62 ± 39.18*	**0.000**
	**AST**	**27.35 ± 8.85**	20.38 ± 8.79*	**0.004**
	**ALT**	**24.35 ± 13.80**	**21.30 ± 10.68**	**0.657**
**50-59 (Non-oil workers, n = 13), (Oil workers, n = 64)**	**AGE**	**53.92 ± 2.78**	**53.23 ± 2.61**	
	**Service period**	**24.67 ± 8.29**	**21.97 ± 5.64**	**0.106**
	**Total bilirubin**	**0.62 ± 0.17**	0.66 ± 0.23*	**0.052**
	**ALK. PHOS**	**69.25 ± 24.09**	125.68 ± 39.08*	**0.000**
	**AST**	**28.02 ± 5.77**	**23.76 ± 18.03**	**0.236**
	**ALT**	**22.37 ± 5.87**	**21.43 ± 9.68**	**0.768**

Multiple Comparisons using Least Significant Difference (LSD) was the statistical method of analysis applied. * indicates statistically significant difference.

In all age groups, while bilirubin was non-significantly higher (P > 0.05) in oil workers, alkaline phosphatase remained very significantly higher (P < 0.01) in oil workers than in non-oil workers. AST and ALT remained consistently insignificantly lower (P > 0.05) in oil workers for all age groups except in age groups 40-49 years where the decrease in AST was significant (P < 0.05) among oil workers.

Finally, the oil workers were also grouped according to their service years or exposure periods (i.e. number of years the worker had been engaged in the work, amounting to his/her cumulative years of exposure to the hazards of his/her workplace) and the studied parameters compared between the various exposure groups of oil workers with those of the non-oil workers, and the results were as presented in Table 5. The results here revealed that, in all exposure groups, alkaline phosphatase was significantly higher (P < 0.05) in oil workers than non-oil workers; bilirubin, though also increased in oil workers for all exposure groups, the increase was significant (P < 0.05) only for exposure periods 6–10, 21–25 and 26–35 years respectively. AST was decreased in all exposure periods of oil work with the decrease being significant (P < 0.05) only for exposure periods 11–15, 16–20 and 26–35 years respectively. On the other hand, ALT was insignificantly lower (P > 0.05) in oil workers for all exposure periods except exposure periods 6–10 and 11–15 years.

**Table 5:** Value ranges and means of liver function parameters studied for the various exposure periods (service years) of the oil workers & control subjects

**Exposure period**	**N**	**Parameter**	**Range**	**Mean**	**P-value**	**Normal range & unit of parameter**
**1-5**	**23**	**Total bilirubin**	**0.5 -1.00**	**0.66 ± 0.14**	**0.134**	**0- 1.00 mg/dl**
**6-10**	**14**		**0.5 -1.20**	**0.71 ± 0.20**	**0.027**	
**11-15**	**128**		**0.4 -1.30**	**0.64 ± 0.18**	**0.123**	
**16-20**	**56**		**0.4 -1.00**	**0.64 ± 0.15**	**0.161**	
**21-25**	**61**		**0.4 -1.30**	**0.69 ± 0.23**	0.011*	
**26-35**	**21**		**0.3 -1.60**	**0.75 ± 0.31**	0.001*	
**Control**	**30**		**0.5-1.00**	**0.58 ± 0.13**		
**Total**	**333**		**0.3-1.60**	**0.65 ± 0.19**		
**1-5**	**23**	**ALK. PHOS**	**82.00 -193.00**	**127.00 ± 32.09**	0.000*	**50-250 U/L**
**6-10**	**14**		**84.00 - 220.00**	**137.50 ± 36.63**	0.000*	
**11-15**	**128**		**58.00 - 270.00**	**129.68 ± 40.17**	0.000*	
**16-20**	**56**		**50.00 - 286.00**	**125.59 ± 43.81**	0.000*	
**21-25**	**61**		**51.00 - 296.00**	**120.51 ± 40.14**	0.000*	
**26-35**	**21**		**61.00 - 163.00**	**114.90 ± 28.14**	0.000*	
**Control**	**30**		**40.00-111.00**	**66.83 ± 18.54**		
**Total**	**333**		**40.00 - 296.00**	**120.86 ± 41.70**		
**1-5**	**23**	**AST**	**8.2 - 40.20**	**21.78 ± 6.88**	**0.101**	**0-45 U/L (M), 0-35 U/L (F)**
**6-10**	**14**		**11.1 - 38.30**	**22.94 ± 8.31**	**0.278**	
**11-15**	**128**		**6.7 - 50.20**	**22.05 ± 8.98**	0.034*	
**16-20**	**56**		**5.8 - 65.00**	**21.79 ± 10.86**	0.045*	
**21-25**	**61**		**6.6 - 140.20**	**22.08 ± 17.94**	**0.055**	
**26-35**	**21**		**8.1 - 39.30**	**18.87 ± 9.12**	0.013*	
**Control**	**30**		**18 - 44.00**	**26.89 ± 6.99**		
**Total**	**333**		**5.8 - 140.20**	**22.26 ± 11.25**		
**1-5**	**23**	**ALT**	**9.1 - 49.70**	**20.93 ± 10.65**	**0.657**	**0-45 U/L (M), 0-35 U/L (F)**
**6-10**	**14**		**10.7 - 42.40**	**23.76 ± 9.66**	**0.685**	
**11-15**	**128**		**7.0 - 86.60**	**24.11 ± 13.36**	**0.422**	
**16-20**	**56**		**6.7 - 52.20**	**21.21 ± 9.36**	**0.665**	
**21-25**	**61**		**4.9 - 48.60**	**19.34 ± 9.03**	**0.235**	
**26-35**	**21**		**6.7 - 37.30**	**20.97 ± 7.75**	**0.674**	
**Control**	**30**		**10.0 - 55.00**	**22.30 ± 10.22**		
**Total**	**333**		**4.9 - 86.60**	**22.15 ± 11.17**		

Multiple Comparisons using Least Significant Difference (LSD) was the statistical method of analysis applied. * indicates statistically significant difference.

Key for letters in Normal Range.

M = Males.

F = Females.

## Discussion

As the data were subjected to statistical analyses using Student’s T-test, the mean values for Total bilirubin and Alkaline phosphatase (ALP) were significantly higher (P < 0.05) in oil than non-oil workers, while Aspartic transaminases (AST) and Alanine transaminases (ALT) were lower, with the decrease in AST being significantly different (P < 0.05) in oil workers than non-oil workers (Table 1). This remained the pattern of presentation even as the study participants were separated according to gender and equivalent sexes also compared using Student’s T-test. The increase observed in the mean values of bilirubin for oil workers over the non-oil workers did not differ significantly (P > 0.05) (Tables 2 and 3). Though the mean values for the parameters were higher in males than females, the increases were not significant in most cases.

When the population was separated into age groups, and their data subjected to Multiple Comparisons using Least Significant Difference (LSD) (Table 4), the findings showed that in all age groups, while bilirubin was non-significantly higher (P > 0.05) in oil workers, alkaline phosphatase remained very significantly higher (P < 0.01) in oil workers than in non-oil workers. AST and ALT remained consistently insignificantly lower (P > 0.05) in oil workers for all age groups except in age groups 40-49 years where the decrease in AST was significant (P < 0.05) among oil workers. As the oil work population was separated on the basis of exposure period and then compared with non-oil work population by the method of Multiple Comparisons using Least Significant Difference (LSD) (Table 5), it was observed that, in all exposure groups alkaline phosphatase was significantly higher (P < 0.05) in oil workers than non-oil workers; bilirubin, though also increased in oil workers for all exposure groups, the increase was significant (P < 0.05) only for exposure periods 6–10, 21–25 and 26–35 years respectively. AST was decreased in all exposure periods of oil work with the decrease being significant (P < 0.05) only for exposure periods 11–15, 16–20 and 26–35 years respectively. On the other hand, ALT was insignificantly lower (P > 0.05) in oil workers for all exposure periods except exposure periods 6–10 and 11–15 years. Thus, from all considerations we have a pattern of consistently significant increase in alkaline phosphatase among oil workers than the control group; consistent increase in bilirubin among oil workers that was significant in some instances and insignificant in other instances; and consistent decrease in AST and ALT among oil workers. Thus, Age and exposure group separations revealed that irrespective of the age of the worker, whatever the effects indicated by these parameters start showing up after the first five years (i.e. exposure period 6-10 years) with a possibility of becoming fully established after the first decade of occupational exposures in this industrial sector (i.e. exposure period 11-15 yrs and above).

To verify the relationships between the parameters under study, data of the entire study participants were jointly subjected to the Pearson’s (2-tailed) correlation analyses, and the results showed that no correlation existed between age and the various parameters except with ALP where there was significant negative correlation (= -0.131, P < 0.017). Similarly, exposure period correlated with no other parameter except ALP, still with negative correlation (=-0.154, P < 0.005). However, there were significant positive correlations between bilirubin and ALP (=0.224, P < 0.000), bilirubin and AST (=160, P < 0.003), bilirubin and ALT (=0.203, P < 0.000). In all instances, there were strong positive correlation among the enzymes: ALP and AST (=0.163, P < 0.003), ALP and ALT (=0.279, P < 0.000), AST and ALT(= 0.545, p < 0.000).

### Liver function profiles: implications for the oil workers

The plasma enzymes including ALP, AST and ALT together with the bilirubins form part of the major functional markers of the liver [49-52]. However, because the liver performs several roles including synthetic, storage, metabolic, excretory and detoxification functions [50-52], it stands out clearly as one organ whose true functional assessment involves the assemblage of a battery of general body functional markers including blood glucose, plasma proteins, plasma lipids and some haematological markers. However, within the confines of the four parameters assayed in this study and presently under consideration- ALP, AST, ALT and bilirubin, though none of these exceeded their individual parametric reference values, the fact that both AST and ALT were consistently lower in the face of a consistently elevated alkaline phosphatase and total bilirubin values in the oil workers in comparison with the referents clearly points to the existence of certain conditions among the oil workers that does not obtain among the non-oil workers.

AST is present in high concentration in cells of cardiac and skeletal muscles, liver, kidney and erythrocytes. Thus, damage to any of these tissues may increase plasma AST levels. In like manner, ALT is present in high concentrations in liver and to a lesser extent in skeletal muscle, kidney and heart [51]. Marked increase of the enzymes (10 to 100 times the upper limit of adult reference range) may be caused by circulatory failure with ‘shock’ and hypoxia, myocardial infarction (particularly for AST) and acute viral or toxic hepatitis, while moderate increase may be caused by [51]:

– Cirrhosis (enzyme levels may be normal or up to twice the upper adult reference limit); infectious mononucleosis (due to liver involvement); cholestatic jaundice (levels may be up to 10 times the upper reference limit); liver congestion secondary to congestive cardiac failure (particularly ALT)

– Malignant infiltration of the liver (particularly for AST and this may be normal or rise to twice the upper reference limit); surgery or extensive trauma and skeletal muscle disease (ALT is much less affected than AST)

– Alkaline phosphatase – a group of enzymes that hydrolyze organic phosphates at high pH, are present in most tissues but are in particularly high concentration in the osteoblasts of bone and the cells of the hepatobiliary tract, intestinal wall, renal tubules and placenta [50,51]. According to these authors, possible causes of raised plasma Alp apart from physiological are:

– Bone disease: rickets and osteomalacia, Paget’s disease of bone (Alp may be very high); secondary malignant deposits in bone; osteogenic sarcoma, only if extensive; primary hyperparathyroidism with extensive bone disease (Alp is usually normal but may be slightly elevated); Secondary hyperparathyroidism.

– Liver diseases: Intra or extra hepatic cholestasis; space-occupying lesions, tumours, granulomas, and other causes of hepatic infiltration.

– Malignancy with bone or liver involvement or direct tumour production. A placental-like (so-called ‘Regan’ Isoenzymes of Alp may occasionally be identified in plasma in patients with malignant disease, especially carcinoma of the bronchus [48,51].

– Leukaemia cells may also leak Alp. A raised plasma (and urinary) level is found in acute monocytic and myeloid leukaemia.

Given the multiple locations of these enzymes, i.e. their presence in many tissues, changes in their plasma activities may reflect damage to any of those tissues, thus allowing room for a needful differential diagnosis. Hence, a careful evaluation of the variously highlighted conditions above is relevant alongside consideration of the plasma bilirubin concentrations. Bilirubin, as a bile pigment, is a product of red blood cells (erythrocytes) broken down by the reticuloendothelial system (RES) found mainly in the spleen. About 80% of bilirubin is derived from haem within the RES, other sources being the breaking down of immature red cells in the bone marrow and of compounds chemically related to haemoglobin such as myoglobin and the cytochromes. Bilirubin is bound to albumin and transported to the liver where it is conjugated, and after conjugation, excreted by active processes into the bile canaculi- a process that is largely dependent on active secretion of bile acids from hepatocytes. Thus, when bile secretion from the hepatocytes into the canaculi is impaired, intrahepatic cholestasis ensues as against extrahepatic cholestasis, which occurs when there is obstruction to the flow of preformed bile through the biliary tract. In these conditions plasma bilirubin levels and alkaline phosphatase activities are increased [51].

In the present study, the increases observed in both ALP and bilirubins were only in relative terms vis-a-vis oil workers and their control counterpart. The mean values of both parameters were still within their normal ranges and for bilirubin in particular, a state of jaundice is not yet reached- a condition of ‘Anicteric’ elevation of Alp without a corresponding increase in AST and ALT. This definitely rules out infective conditions such as viral hepatitis (as AST and/or ALT ought to have increased correspondingly) [51] but certainly not toxic hepatitis, which according to Davis [53], is possible as a result of enzyme induction in the face of certain environmental agents such as aromatic hydrocarbons (and these are superabundantly present in petroleum products), some food additives and some pesticides, which according to Conney [54] have enzyme-inducing properties. Again, chronic exposure to enzyme-inducing agents, according to Stamp et al. [55], may lead to osteomalacia (one of the possible causes of increases in plasma Alp concentration) as a result of diversion of vitamin D metabolism to biologically inactive metabolites. Also, many carcinogens and some hepatotoxins exert their toxic effects via metabolites, and enzyme induction potentiates the activation of these compounds and therefore their toxicity [56,57]. So, the question is: What does Anicteric elevation of AlP indicates for the oil work population?

The total alkaline phosphatase assayed consisted of several isoenzymes that can only be distinguished electrophoretically (not done in this study). These include isoenzymes from cells of bone, liver, biliary tract, intestine and placenta [49-51]. It is excessively rare to find all five fractions together in the same sample of serum. Placental AlP is present only in pregnant women (this is not applicable in this study). The presence of intestinal AlP seems related to liver cirrhosis and also the ABO secretor status, higher levels being found in individuals of blood groups O and B [58,59] though the concentration in the intestinal tissue is not related to blood groups [60]. The presence of both liver and bile isoenzymes in anicteric patients point to an impairment of the bile flow termed ‘focal block’. The macromolecular bile components are refluxed back into the blood, which they cannot leave again. The low molecular weight components such as bilirubin are however, again extracted from the blood and secreted in zones where the bile flow is not blocked [61]. The most frequent cause of this type of obstruction is the presence of malignant primary or secondary tumour in the liver [49].

Another interesting change in serum alkaline phosphatase in malignancy is the appearance in the serum of an isoenzyme that cannot be distinguished from placental AlP (i.e. placental-like Alp) called ‘Regan isoenzyme’ after the patient in whom it was first demonstrated. Apart from this patient with bronchial carcinoma (the isoenzyme was found in both the serum and tumour), it was found in the serum of 27 out of 500 patients with various malignant diseases [59]. Warnock and Reisman [62] found a similar enzyme in extracts of eight out of 10 hepatomas and also in the serum of some of these patients. In all these instances as in this study, total serum alkaline phosphatase was increased.

Although serum electrophoresis was not done in this study to distinguish the isoenzyme fractions of the total AlP (elevated in the oil workers over their control peers) and possibly establish or rule out other conditions including presence of tumours or carcinomas, it is believed that a reasonable pointer has been shown as to the possible conditions indicated by Anicteric significant rise in alkaline phosphatase among oil workers. Based on these submissions, it could be said that part of the health risks associated with occupational exposures in the Oil and Gas industry are among others, the possible development of bone diseases with increased osteoblastic activity, liver diseases with involvement of the biliary tracts, toxic hepatitis, liver cirrhosis, malignancies of various body sites including bronchial carcinoma, hepatoma and leukaemia, etc. In fact, apart from the various degrees of accidents and injuries previously reported among these same petroleum refining and distribution workers [63], results also indicated that some of the above highlighted conditions including liver diseases, made reckonable contributions to the morbidities and mortalities recorded in this industry [64], thus suggesting the presence of organs/systems toxic agents in this work environment. Serum alkaline phosphatase electrophoreses study is therefore highly warranted in a further study of these oil refining and distribution workers to establish the actual isoenzyme fraction of alkaline phosphatase responsible for the increasing pattern observed for this enzyme among this segment of workers, and thus establish on a firmer basis some or all of these possible health risks among the oil workers.

Just like in our own study, previous works relating to occupational exposures had recorded variable levels of these same liver markers. Contrary to the findings in the present study, elevated levels of AST and ALT were reported for those occupationally exposed to toxic substances, indicating susceptibility to Anicteric hepatitis [63,64]. The study of Dioka et al. [65] revealed that while ALT and AST activities were elevated in occupationally exposed artisans than in controls, ALP activity was significantly lower in the exposed subjects than in controls. Waldman and Borman [66] had earlier reported similar findings of increased serum ALT and AST activities. Studies by Kapaki et al. [67] showed an ALT elevation in taxi drivers, while activities of both ALT and AST were elevated in gas station workers.

Similarly, other animal studies produced variable results in enzyme levels following exposure to petroleum products. In fish, serum levels of AST, ALT and ALP were increased following hepatocellular damage resulting from exposure to 2, 3, 4-triaminoazo benzene [68]. Other studies also indicated increase in the activities of the liver enzymes following liver damage in fish and albino mouse exposed to toxic substances [69-71]. Chu et al. [72] noted an increase in levels of serum aspartate aminotransferase (AST) in male rats and alkaline phosphatase (ALP) in female rats exposed to petroleum hydrocarbon, whereas administration of 200, 400, and 800 mg/kg of Bonny light crude oil in the study of Orisakwe et al. [34] caused a significant dose-dependent increase in AST and ALT, but a significant decrease in the alkaline phosphatase (ALP) level compared to the control. Thus, administration of the bonny light crude oil produced a dose-dependent damage of the liver cells.

A three months study of biochemical changes in the serum and liver of albino rats chronically administered with 5.0gkg^-1^, 7.5gkg^-1^ and 15.0gkg^-1^ of gasoline, kerosene, and crude petroleum (bonny light) respectively showed that the petroleum samples caused biochemical changes in the serum and liver parameters [38]. Thus, AST, ALT and ALP showed dose-dependent increase in levels from first month to the 3^rd^ month for all the petroleum samples, with substantial to marginal increases that are significantly different from the control (p < 0.05) following administration of various combinations of the samples. Elevations of these enzymes reflect presence of some inflammatory disease or injury to the liver, while the maximum ALP activity (more than two fold) obtained for gasoline and kerosene for the third month showed the possibility of hepatocellular damage.

Histopathological study of Liver of rats administered with diesel fuel showed that this substance induced a dose-dependent effects ranging from narrowing of sinuses (LD_50_) to severe hepatocellular necrosis (LD_100_), thus qualifying to be included as a hepatotoxin [36]. Hepatotoxins are defined as agents that cause liver injury after a relatively short period, and which may cause liver cell necrosis alone or with altered enzyme activity and biliary tract dysfunction [73]. However, another study involving exposure of male and female mice to various doses (up to 2056 ppm) of PS-6 blend gasoline vapour 6 hr/day, 5days/week up to 13 weeks, showed that though the leaded gasoline increases hepatocellular proliferation in mice, it did not significantly affect serum ALT and AST activity or cause any histological changes in the liver [74]. Mechanism of gasoline toxicity or indeed other toxic substances may include inactivation of antioxidants such as Glutathione (GST), and this may explain the low activity of GST in gasoline-injected rats in the study of Ayalogu et al. [38]. Chiapotto et al. [75] had also reported inactivation of GST by different concentrations of acetaldehyde. Although GST assay was not done in the present study, these reports have shown that enzyme pattern in the serum or plasma reflects the physiological state of the organ.

## Conclusion

The study revealed that though the levels of the assayed hepatic biochemical markers were still within the parametric reference ranges as at the time of this study, however, relative to the non-oil work referents that served as the control group, changes observed in the biochemical markers among the oil workers suggested that petroleum refining and distribution work environment is furnished with some hepatoxic substances and that anicteric hepatoxicity is a potential health effect of long term occupational exposures in the petroleum refining and distribution industry in Nigeria, hence, long-serving staff might be victims of anicteric toxic hepatitis.

Our inability to obtain and adjust data for the smoking habit of the study participants was a major deficiency of the study, which needs to be taken care of in any future study of these industrial workers. However, despite this shortcoming, the findings offer far reaching contributions in the area of occupational and environmental toxicology, given that most of what was known of petroleum toxicology in Nigeria were based on studies using animal models. To the best of our knowledge, this is the first study on this thematic area conducted directly on humans in Nigeria. While earlier studies have reported the effects of petroleum exposures in animal models for extrapolation to humans, our study was carried out using direct human biological samples. Thus, the results give a more assured situation than an extrapolated result, which might be affected by species differences

These study findings have important applications, since it provided the management of the establishments within this industrial sector with data-based evidence for upgrading the industrial/occupational health measures aimed at improving the health of their workers and eventually their productivity. In this regard, the study results indicated a need for frequent environmental and biological monitoring to ensure a safer and healthier workplace and workforce respectively in this very critical sector of the Nigerian economy. Again, in the realms of forensic toxicology, the study findings also offer immense benefit, since it provided scientific evidence for resolution of health compensation litigations, particularly those involving occupationally-facilitated hepatic diseases conditions. Finally, through this study, hepatic health data on petroleum refining and distribution industry workers have been provided, and this marks an important response to the calls for data by Loewenson (19) regarding industrially furnished exposures and their health effects, and also by Scala (20) for toxicological data on the petroleum/petrochemical industry. The situation as it affects other organs/systems still needs to be addressed, and remains the focus of our on-going studies.

## Competing interests

The authors declare that they have no competing interests.

## Authors’ contributions

TINE conceived and designed the study, collected, analyzed and interpreted the data, and also drafted the manuscript. ANE and OEO assisted in drafting the manuscript, MOEI supervised the study while FOUO and HCN co-supervised the study. The authors have read and approved the final manuscript.
